# Proteomic approach with LCMS-IT-TOF identified an increase of Rab33B after transient focal cerebral ischemia in mice

**DOI:** 10.1186/2040-7378-2-20

**Published:** 2010-11-23

**Authors:** Kana Hyakkoku, Junya Hamanaka, Kazuhiro Tsuruma, Masamitsu Shimazawa, Hideaki Hara

**Affiliations:** 1 Molecular Pharmacology, Department of Biofunctional Evaluation, Gifu Pharmaceutical University, 1-25-4 Daigaku-nishi, Gifu 501-1196, Japan

## Abstract

**Background:**

Several proteins are known to be markedly expressed in the brain during cerebral ischemia; however, the changes in protein profiles within the ischemic brain after an ischemic insult have not been fully elucidated. We studied the changes in the ischemic brain proteome after focal cerebral ischemia, induced by middle cerebral artery occlusion (MCAO) in mice.

**Methods:**

LCMS-IT-TOF mass spectrometry was used to detect the changes in ischemic brain protein patterns after MCAO. We evaluated the protein expression detected in the ischemic area, by western blotting and immunohistochemistry.

**Results:**

Nine unique proteins were identified from the ischemic area at 10 h after ischemic insult. Among these proteins, we focused on Rab33b, a member of RAS oncogene family and we found that Rab33b was up-regulated in the ischemic striatum and the number of Rab33B-positive cells increased in a time-dependent manner. Rab33B colocalized with Iba-1 positive microglia in the ischemic area.

**Conclusion:**

These findings suggest that LCMS-IT-TOF is useful for identifying changes in proteins after cerebral ischemia and that Rab33B is partially related to the pathogenesis of transient cerebral ischemia in mice.

## Background

Stroke is the third most common cause of death, after heart attack and cancer, and it has profound negative social and economic effects. The current treatment for complete stroke is only partially successful at reversing neurodegeneration and restoring premorbid function. Since stroke is one of the principal etiologies for neurological sequelae and/or death, it is very important to understand both its pathologic mechanisms and any effective treatments. Understanding the cellular mechanisms of ischemia-reperfusion injury is of great importance for stroke therapy; however, the precise mechanisms still remain unclear. The identification and characterization of the ischemic area are valuable and useful for understanding the pathogenesis and for establishing new therapeutic strategies to treat or prevent brain ischemia.

Proteome analysis, an exhaustive analytical technique for analyzing proteins, is primarily conducted using ESI-LCMS or MALDI-TOFMS. Many proteins and peptides are extremely minute in quantity, requiring the enhanced sensitivity provided by mass spectrometers. Shimadzu's new LCMS-IT-TOF is a novel hybrid mass spectrometer that is applicable to a wide range of bioanalytical research, including biomarker discovery, metabolite identification, and drug development, among others. Coupling atmospheric pressure ionization with Ion-Trap (IT) and Time-of-Flight (TOF) technologies, the LCMS-IT-TOF delivers high mass accuracy and high mass resolution (10,000 at 1000 m/z) independent of MS mode. The LCMS-IT-TOF allows more qualitative information about a sample to be collected in a single run. This enables researchers and scientists to: 1. elucidate structures of new molecules; 2. identify impurities and contaminants; and 3. analyze metabolites and biomarkers to evaluate biological pathways. The LCMS-IT-TOF has been designed to maximize sensitivity and selectivity by optimizing the ion transport to the TOF analyzer and by redefining the capability of the quadrupole ion trap. The ion trap is used to focus ions prior to ejection into the TOF as well as to support MS^n ^analysis with effective precursor ion selection capabilities (resolution > 1,000 at 1,000 m/z).

The quest for biological markers of disease is a challenge for investigators in many fields, and this is certainly the case in the study of neurologic disease. One of the goals of proteomics is to characterize cellular proteins, secreted proteins and peptides, and proteolytic fragments for use as potential biomarkers. The objective of the present study was to identify ischemia-related proteins using the new LCMS-IT-TOF to analyze the chronological change of protein peaks following ischemia/reperfusion.

## Methods

### Animals

The experimental designs and all procedures were in accordance with the U.S. National Institutes of Health Guide for the Care and Use of Laboratory Animals and Animal Care Guidelines issued by the Animal Experimental Committee of Gifu Pharmaceutical University. All experiments were performed using male ddY mice (4-5 weeks old, Japan SLC, Ltd., Shizuoka, Japan). Every effort was made to minimize the number of animals used and their suffering.

### Drugs

Formic acid (HCOOH) and *trifluoroacetic acid *(TFA) were purchased form Wako pure chemical (Osaka, Japan). HPLC-grade ACN (CH3CN) and water were also obtained from Wako pure chemical. A Liquid Tissue MS Protein Kit was obtained from Expression Pathology Inc. (Gaithersburug, MD, USA). Pentobarbital sodium and isoflurane were purchased from Nissan Kagaku (Tokyo, Japan) and Merck Hoei Ltd. (Osaka, Japan), respectively.

### Focal cerebral ischemia model in mice

Anesthesia was induced using 2.0 to 3.0% isoflurane and maintained using 1.0 to 1.5% isoflurane (both in 70% N_2_O/30% O_2_) by means of an animal general anesthesia machine (Soft Lander; Sin-ei Industry Co. Ltd., Saitama, Japan). Body temperature was maintained at 37.0 to 37.5°C with the aid of a heating pad and heating lamp. After a midline skin incision, the left external carotid artery was exposed, and its branches were occluded [1,2]. An 8-0 nylon monofilament (Ethicon, Somerville, NJ, USA) coated with a mixture of silicone resin (Xantopren; Bayer Dental, Osaka, Japan) was introduced into the left internal carotid artery through the external carotid artery stump so as to occlude the origin of the middle cerebral artery. Then, the left common carotid artery was occluded. After 2 h of occlusion, the animal was reanesthetized briefly and reperfusion initiated via withdrawal of the monofilament. After surgery, the mice were kept in the preoperative condition (24 ± 2°C) until sampling. To confirm the induction of MCAO, a laser-Doppler flowmetry (Omegaflow flo-N1; Omegawave Inc., Tokyo, Japan) measured the regional artery blood flow (rCBF) in the MCA territory from the temporal bone surface. Mice that did not demonstrate a significant reduction just before reperfusion (to less than 40% that of the contralateral rCBF values) were excluded.

### Tissue micro dissection

The left ventricle was perfused with 4% paraformaldehyde in 0.1 M phosphate buffer (PB; pH 7.4). Brains were removed after 15 min perfusion fixation at 4°C, then immersed in the same fixative solution overnight at 4°C. They were then immersed in 25% sucrose in 0.1 M PB for 24 h, and finally frozen in liquid nitrogen. Coronal sections (14 μm thick) were cut on a cryostat at -20°C, and the ischemic core area and a non-ischemic area (2 mm × 2 mm) were excised and placed in clear 1.5 ml tubes.

### Protein Extraction

Micro dissected specimens were processed by reagents according to the manufacturer's recommendations (Expression Pathology Inc., Rockville, MD, USA). Briefly, material was suspended in 20 μl of reaction buffer, incubated at 95°C for 90 min, then cooled on ice for 2 min, at which time 1 μl of trypsin was added followed by incubation at 37°C for 18 h. Dithiothreitol was added to a final concentration of 10 mM, and the sample were heated for 5 min at 95°C to reduce cysteine residues. Finally, Liquid Tissue MS Protein Kit extracts were desalted with C-18 Zip Tip microcolumns (Millipore, Bedford, MA, USA), lyophilized, and resuspended in a minimal amount of 0.1% formic acid in 2% ACN.

### NanoRPLC-MS/MS Analysis

NanoRPLC was performed using a DiNa-2A nanoLC system (KYA Technologies, Tokyo, Japan) coupled online to a LCMS-IT-TOF (Shimadzu, Kyoto, Japan). NanoRPLC separation was performed using a Pico Frit Beta Basic C18 column (New Objective, Woburn, MA, USA) at a constant flow rate of 300 nl/min. After injection of 1 μl of sample, peptides were eluted using gradients of 5-40% solvent B (0.1% formic acid in 80% ACN)/0-30 min, 40-100% solvent B/30-40 min, and 100-100% solvent B/40-60 min. LCMS-IT-TOF was operated in the data-dependent MS/MS mode. The heated capillary temperature and electrospray voltage were set at 200°C and 2.3KV, respectively. Data were collected at scan ranges of 400-1500 for MS and 50-1500 for MS/MS.

### MS/MS spectra analysis

MS/MS spectra were searched against the NCBI database using Mascot (Matrix Science Ltd, London, UK) with a peptide mass tolerance of ± 0.05 Da and a fragment mass tolerance of ± 0.05 Da.

### Western blotting

**Figure 1:**
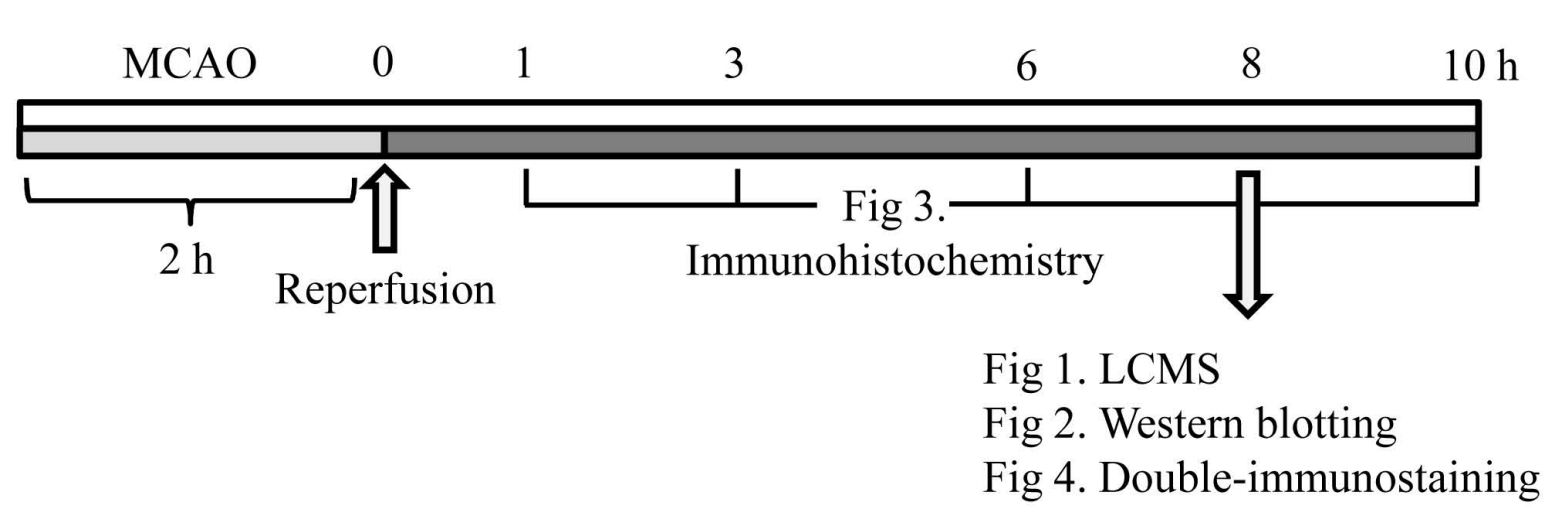
**Protocol of all experiments**. The sampling time are mentioned. See in method section.

Mice were deeply anesthetized and decapitated at 8 h after reperfusion (Figure 1), brains were quickly removed, and the left hemispheres were cut into 3 mm coronal sections (between 4 and 7 mm from the frontal forebrain). Brain samples were divided into cortex and striatum and each sample was homogenized in 10 ml/g tissue ice-cold lysis buffer [50 mM Tris-HCl (pH8.0) containing 150 mM NaCl, 50 mM EDTA, 1% Triton X-100, and protease/phosphatase inhibitor mixture] and centrifuged at 14,000 × g for 40 min at 4°C. An aliquot of 5 μg of protein was subjected to 10% sodium dodecyl sulfate-polyacrylamide gel electrophoresis and then separated proteins were transferred onto a polyvinylidene difluoride membrane. For immunoblotting, the following primary antibodies were used: mouse anti-Rab33B monoclonal antibody (1 μg/mL: Frontier Science, Hokkaido, Japan), monoclonal anti-β-actin (1:1000 dilution: Sigma-Aldrich Co., St. Louis, MO, USA). The secondary antibody was anti-mouse HRP-conjugated IgG (1:2000 dilution, Pierce Biotechnology, Rockford, IL, USA). The immunoreactive bands were visualized using SuperSignal West Femto maximum sensitivity substrate (Pierce Biotechnology). The band intensity was measured using a Lumino imaging analyzer (LAS-4000: Fuji Film, Tokyo, Japan).

### Immunohistochemistry

At 1, 3, 6, and 10 h after the 2 h ischemia-reperfusion treatment (Figure 1), mice were injected with sodium pentobarbital (Nembutal; 50 mg/kg, i.p.), then perfused through the left ventricle with 4% paraformaldehyde in 0.1 M phosphate buffer (PB; pH 7.4). Brains were removed after 15 min perfusion fixation at 4°C, then immersed in the same fixative solution overnight at 4°C. They were then immersed in 25% sucrose in 0.1 M PB for 24 h, and finally frozen in liquid nitrogen. Coronal sections (14 μm thick) were cut on a cryostat at -20°C and stored at -80°C until use.

Sections were rinsed three times in PBS, incubated in 3% H_2_O_2 _in PBS for 30 min, then placed in PBS and blocked with 1% mouse serum for 30 min. A mouse monoclonal antibody against Rab33b (3 μg/mL: Frontier Science) was applied to sections overnight at 4°C. Secondary antibody (M.O.M. biotinylated anti-mouse) was applied for 10 min. The avidin/biotinylated horseradish peroxidase complex (ABC Elite kit; Vector Laboratories, Burlingame, CA, USA) was applied for 30 min, and the sections were allowed to develop the chromogen in 3,3-diaminobenzidine nickel solution for 2 min. Rab33B-stained cells were counted in the ischemic area.

### Double-immunostaining

For immunohistochemistry, mice were anesthetized with sodium pentobarbital and their brains were perfused with 4% paraformaldehyde at 8 h after the 2 h ischemia-reperfusion treatment (Figure 1). The perfused brains were dissected out, fixed in 4% paraformaldehyde for overnight, and frozen. Coronal brain sections (14 μm) were cut on a cryostat. For immunofluorescent double staining, sections were incubated overnight at 4°C with the primary antibodies: anti-Rab33B antibody (3 μg/mL: Frontier science), or anti- Iba-1 antibody (1:500 dilution: Wako pure chemical). Then, they were incubated for 3 h with Alexa Fluor 488 F (ab')_2 _fragment of goat anti-rabbit IgG (H+L) antibody, Alexa Fluor 546 F (ab')_2 _fragment of goat anti-mouse IgG (H+L) antibody.

### Cell counting

To quantify Rab33B-positive cells after ischemia-reperfusion, the number of Rab33B-positive cells in the ischemic striatum; two areas, the superior and inferior areas were counted in a high-power field (×200) randomly chosen in a section through the anterior commissure.

### Statistical analysis

Data are presented as the means ± S.E.M. Statistical comparison were made using a one-way ANOVA followed by Student's *t*-test or Dunett's test using Statview version 5.0 (SAS Institute Inc., Cary, NC, USA), with p < 0.05 considered statistically significant.

## Results

### NanoRPLC-MS/MS Analysis

**Figure 2:**
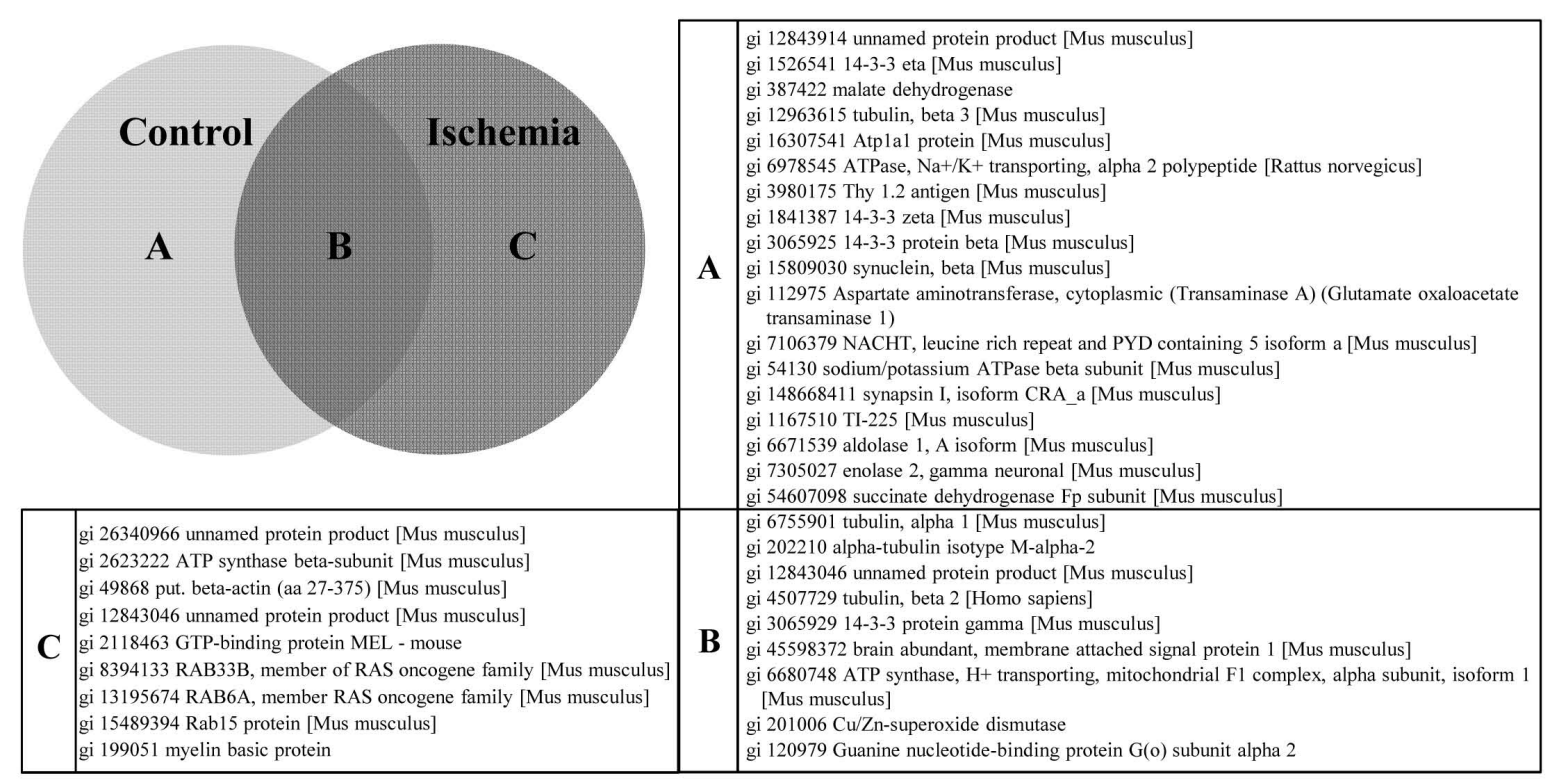
**Proteins identified with NanoRPLC-MS/MS Analysis**. (A) 18 proteins identified from control group only. (B) 9 proteins identified from both control and ischemia group. (C) 9 proteins identified from ischemia group only.

In the control group, 27 proteins were identified from NanoRPLC-MS/MS analysis (Figure 2A and 2B). In an ischemia group at 8 h after 2 h ischemia-reperfusion (Figure 1), NanoRPLC-MS/MS analysis etected 18 proteins from the ischemic area (Figure 2B and 2C). From the NanoRPLC-MS/MS analysis, we identified nine unique proteins that were detected only in the ischemic area (Figure 2C).

### Western blotting for Rab33B

**Figure 3:**
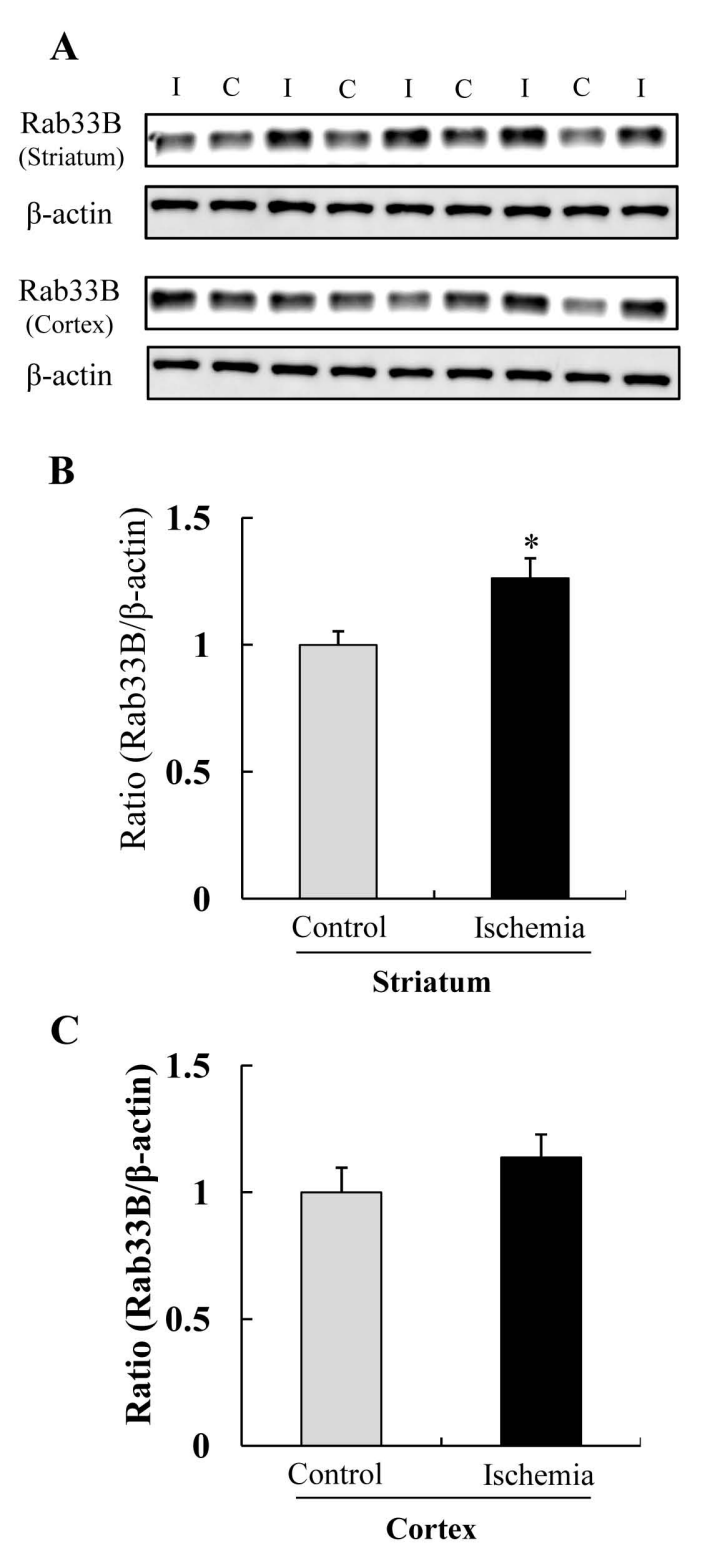
**Western blot analysis of Rab33B in ischemic area at 8 h after 2 h ischemia -reperfusion**. (A) Representative band image showed Rab33B expression of ischemia (I) group and control (C) group. (B) and (C) Rab33B expressions were quantified by densitometry and corrected by reference to β-actin. (* P < 0.05, *t*-test) Data are shown as mean ± S.E.M. (n = 4 or 5).

Of these nine unique proteins (Figure 2C), we focused on RAB33B, a member of the RAS oncogene family. First, we examined whether Rab33B was up-regulated in an ischemic group by western blotting. Rab33B was increased in the ischemic striatum, but not in the ischemic cortex (Figure 3A). In quantitative analysis, Rab33B was present at significantly greater levels in the ischemic striatum than in the control striatum (Figure 3B), but no difference was noted between the ischemic cortex and the control cortex (Figure 3C).

### Expression of Rab33B after ischemia

**Figure 4:**
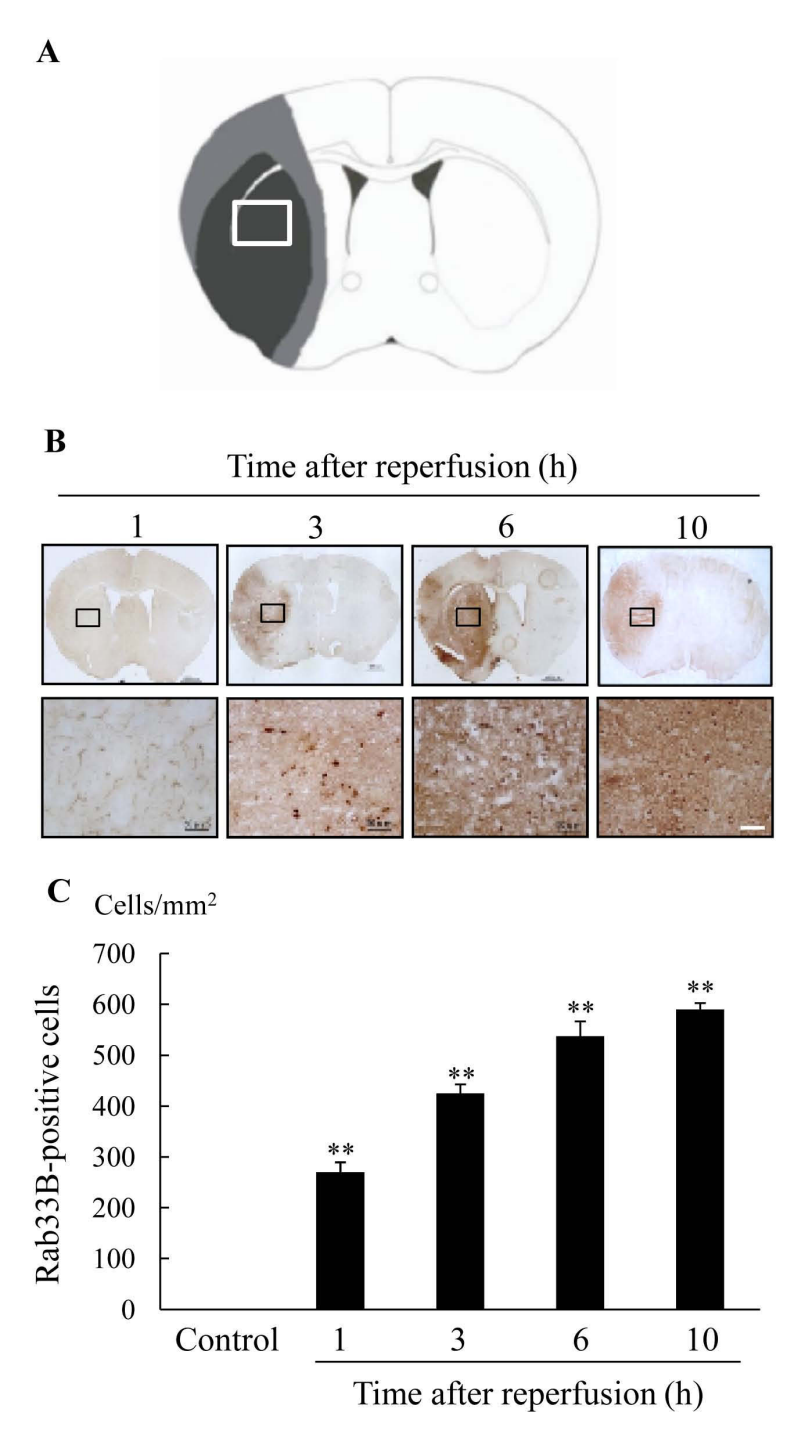
**Change in levels of Rab33B expression in the ischemic area after 2 h ischemia-reperfusion**. (A) Schematic drawing showing brain regions at 0.4-1.0 mm anterior to bregma (through the anterior commissure). (B) Immunostaining for Rab33B at 1 h, 3 h, 6 h, and 10 h after reperfusion in mice. Scale bar = 50 μm. (C) The number of Rab33B-positive cells in the ischemic striatum. Values are shown the number of Rab33B-positive cells/mm^2^. Data are shown as mean ± S.E.M. (n = 4). ** P < 0.01 vs. control (Dunnett's test).

Next, we investigated changes in Rab33B expressions at 1, 3, 6, and 10 h after reperfusion by immunohistochemical analysis (Figure 4B). Quantitative analyses of Rab33B-positive cells are shown in Figure 4C. In the ischemic area, the number of Rab33B-positive cells was increased in a time-dependent manner from 1 h to 10 h following the 2 h ischemia-reperfusion treatment. No Rab33B-positive cells were detected in non-ischemic areas.

### Localization of Rab33B after ischemia

**Figure 5:**
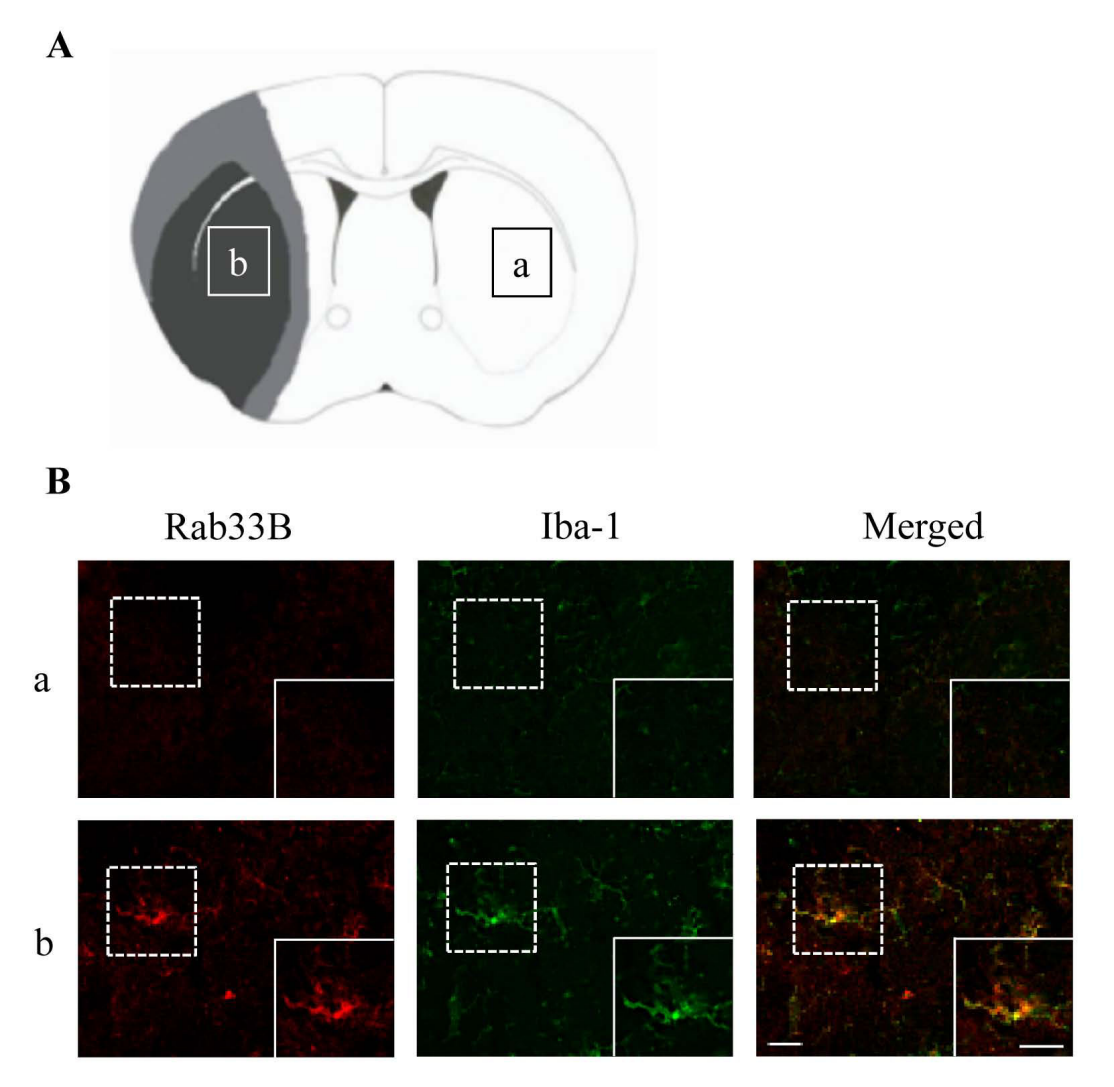
**Localization of Rab33B in ischemic area.** (A) Schematic drawing showing brain regions at 0.4-1.0 mm anterior to bregma (through the anterior commissure): Measurement areas (a and b) in figure 5A in the contralateral non-ischemic and ischemic core, respectively. (B) Double-immunostaining of Rab33B and Iba-1. Rab33B expressed in microglia at 8 h after the ischemia reperfusion. (a) contralateral side (non-ischemic area). (b) ipsilateral side (ischemic area). Scale bar = 20 μm.

To identify Rab33B-positive cells, double immunofluorescence was performed for Rab33B and Iba-1 which is the marker of activated microlgia at 8 h after the 2 h ischemia-reperfusion. Iba-1-Positive microglias were expressed with Rab33b together in ischemic striatum (Figure 5B-b), but not in non-ischemic area (Figure 5B-a).

## Discussion

In the present study, we identified ischemia-related proteins using an LCMS-IT-TOF to analyze the chronological changes in protein peaks following ischemia/reperfusion treatments in mice. LCMS analysis is the useful instrument which is able to detect the candidates of the protein markers for cerebral ischemia. For analyzing the peptides by LCMS, peptides which were obtained by extracting proteins from tissue samples and trypsin digestion were used. There are differences between control and ischemia brain groups in the amount of protein and peptide. And the LCMS detects from the peptide fragments in decreasing order. In the present study, beta-actin of house-keeping gene was detected in only ischemia group due to their differences. The beta-actin was expressed in both ischemia group and control groups, and the expression level was no significant change (Figure 3A). Therefore, the beta-actin may play a role as a house-keeping gene in the present study.

We found an up-regulation of nine unique proteins in the ischemic brain areas. Among these proteins, we identified three Rab proteins. In the Rab family, the past reports concerning Rab33B were few and its function is not well known. Therefore, we focused on Rab33B in the present study. The Rab family of proteins is a member of the Ras superfamily of monomeric G proteins [3]. At least 60 Rab genes are found in the human genome and are widely distributed over the human chromosomes [4]. Rab GTPases have emerged as central regulators of vesicle budding, motility, and fusion and a number of these proteins are conserved from yeast to humans. Like other regulatory GTPases, the Rab proteins switch between two distinct conformations, one GTP-bound and the other GDP-bound. The GTP-bound conformation is generally regarded as the 'active' form, as it is the one that interacts with downstream effector proteins [5,6].

In the present study, we focused on Rab33B, which was identified in ischemic areas by proteome analysis. Rab33B is one of two members of the Rab33 subfamily. In contrast to its neural and immune cell homolog, Rab33B is ubiquitously expressed in mammalian tissues and is localized to the medial golgi [7]. Rab33B has been described as a Golgi-resident protein which is involved in Golgi-to-endoplasmic reticulum transport and in modulation of autophagy [8-10].

Autophagy is an intracellular pathway that is activated in response to cell stress. It is a phenomenon in which the cytoplasmic organelles in the cell are engulfed by double membrane vesicles, the autophagosomes, and are delivered to the lysosomes. There, the organelle proteins are broken down by lysosomal proteases and the resulting amino acids are recycled back into the cell machinery to aid in cell survival [11,12]. A number of key autophagy (Atg) proteins are apparently involved in this process [10,13]. Rab33B directly interacts with one of these, Atg16L, in a GTP-dependent manner. Therefore, activation and inactivation of Rab33B can modulate autophagy [7,8]. Autophagy appears to be a vital event in the development of the central nervous system [14,15] and is also constitutively active in healthy neurons, where it aids in survival [16].

In the present study, we examined whether Rab33B colocalized with LC3 of autophagy marker. However, we could not detect the expression of LC3 in ischemic area at 8 h after 2 h ischemia-reperfusion (data not shown). It is reported that the autophagy was detected in ischemic area with a peak at 1 day after the transient MCAO [17]. In the present study, we focused on the expressions of the ischemia related proteins at early phase after ischemia-reperfusion. Further studies are needed to clarify the involvement of Rab33B to autophagy after cerebral ischemia, especially later phase than the present study.

Microglia, which is one of the glial cells, is approximately 20% of the total glial cell population within the brain. Unlike astrocyte, individual microglias are distributed in large non-overlapping regions throughout the brain and spinal cord [18]. Activation of microglia commonly occurs in the early response of the CNS to a wide variety of pathological stimuli, such as neurological degeneration, inflammation, and ischemia [19-21]. It is unclear that microglias are necessarily damaged following brain ischemia, but recent evidences suggest that activated microglia may contribute to the injury. Some neuroprotective drugs, such as minocycline and edravone, improved the stroke outcomes by inhibiting the microglial activation [22-24]. Microglia exerts a cytotoxic function by releasing reactive oxygen species, nitric oxide, and inflammatory cytokines, which trigger the neuronal damage [25-27]. Several studies have suggested that microglia may be rapidly and time-dependently activated after ischemia, and the microglial activation may reflect the extent of severity of ischemic injury. In the present study, an up-regulation and a time-dependent increase in Rab33B was observed in ischemic striatum (primarily in the ischemic core area) and was colocalized with Iba-1 positive activated microglia. Taken together, Rab33B may contribute to the neuronal cell death in the ischemic core area.

## Conclusions

These findings suggest that Rab33B is involved in the mechanism of pathogenesis of transient cerebral ischemia in mice and that it may be contribute to the neuronal cell death. Thus, control of the Rab33B gene and/or protein after ischemia may prove to be a useful strategy for therapeutic treatment of ischemic stroke. Further studies are needed to clarify the function of Rab33B in cerebral ischemia.

## Competing interests

The authors declare that they have no competing interests.

## Authors' contributions

HH participated in the design of the study. KT and MS analyzed the data. KH and JH performed the study. KH, JH and HH wrote the paper. All authors read and approved the final manuscript.
